# Evaluating the Use of Optical Coherence Tomography in Optic Neuritis

**DOI:** 10.1155/2011/148394

**Published:** 2011-03-22

**Authors:** Fiona Costello

**Affiliations:** Departments of Clinical Neurosciences and Surgery, Hotchkiss Brain Institute, University of Calgary, Calgary, AB, Canada T2N 2T9

## Abstract

Optic neuritis (ON) is an inflammatory optic nerve injury, which is strongly associated with multiple sclerosis (MS). Axonal damage in the optic nerve manifests as retinal nerve fiber layer (RNFL) deficits, which can be readily quantified with optical coherence tomography (OCT). The RNFL represents the most proximal region of the afferent visual pathway; and, as such, is a unique region of the central nervous system (CNS) because it lacks myelin. Changes in retinal integrity can be correlated with reliable and quantifiable visual outcomes to provide a structural-functional paradigm of CNS injury. Because the eye provides a unique “view” into the effects of CNS inflammation, the ON “system model” may provide greater understanding about disease mechanisms, which underpin disability in MS. This review addresses the applications of OCT in study of ON patients, with specific reference to the published reports to date. The future role of OCT is discussed, both in terms of the potential gains and certain challenges associated with this evolving technology.

## 1. Optic Neuritis: An Overview

Optic neuritis (ON) is an inflammatory optic nerve injury, which causes subacute onset vision loss in children and young adults. Much of our understanding regarding the clinical presentation of ON has been derived from the Optic Neuritis Treatment Trial (ONTT) [1]. This randomized, placebo-controlled, multicenter trial compared the visual benefits of treatment with either intravenous methylprednisolone (250 mg every 6 hours for 3 days followed by oral prednisone (1 mg/kg per day) for 11 days), oral prednisone (1 mg/kg per day for 14 days), or oral placebo (for 14 days) in 457 patients with acute ON [1]. From the ONTT, we learned that most ON patients are young (mean age 32 years) Caucasian (85%) women (77%) [1]. Over ninety percent of ON patients report pain at the onset of vision loss [1] which is often characterized as an “ache” made worse with eye movements. Vision loss is generally acute, to sub-acute in onset progressing over a period of hours to days. The severity of vision loss may range from mild (Snellen equivalent of 20/20 vision) to no light perception (NLP) [2]. Dyschromatopsia or decreased color vision is quite common [2], and this finding can help localize the diagnosis in patients with relatively mild visual acuity deficits. Patients with unilateral ON often manifest a relative afferent pupil defect, unless there is coexisting optic nerve damage in the contralateral eye [2]. Visual field defects in ON correspond to the topography of the retinal nerve fiber layer (RNFL) and may be arcuate, altitudinal, or cecocentral in shape. In cases of retrobulbar ON the fundus examination is initially normal, whereas patients with anterior ON or “papillitis” may manifest optic disc swelling [2, 3]. Atypical fundus findings in ON patients include severe optic disc edema, peripapillary hemorrhages, or retinal exudates [4]. Clinical features which are not typical of ON should prompt an investigation for other diagnoses including ischemic, compressive, infiltrative, toxic-metabolic, and inflammatory optic neuropathies.

The majority of ON patients recover vision over a period of weeks [1–3], during which time optic disc pallor may evolve as a “footprint” of the previous inflammatory injury. Yet, even in patients who recover 20/20 vision in their affected eye, persistent visual problems (fatigue-induced vision loss; altered motion and depth perception; loss of contrast sensitivity) are common. Patients with previous ON frequently describe transient vision loss with increased body temperature, which is known as “Uhthoff's” phenomenon [2]. Because axonal damage is an early manifestation in demyelinating plaques of multiple sclerosis (MS) patients [5], persistent visual deficits after ON may be a consequence of prior demyelination and/or permanent axonal damage in the anterior visual pathway.

## 2. Exploring the Link between Optic Neuritis and Multiple Sclerosis

There is a strong association between ON and MS, such that approximately 20% of patients experience ON as their initial demyelinating event, and 30–70% of MS patients develop ON during the course of their disease [6, 7]. Many patients who present with ON as a clinically isolated syndrome (CIS) demonstrate evidence of disseminated central nervous system (CNS) inflammation on their baseline magnetic resonance imaging (MRI) study (50% to 70%) and harbor abnormal cerebrospinal fluid (CSF) constituents (60 to 70%), which increase their future risk of MS [2, 4, 8–11]. After 15 years, 72% of patients in the ONTT who had one or more white matter lesions on their baseline MRI developed clinically definite MS (CDMS) as compared to only 25% of patients with no MRI lesions [10]. 

## 3. Optic Neuritis: A System Model of Multiple Sclerosis?

Vision loss is both prevalent and relevant in MS patients. Because the visual system is a functionally eloquent region of the CNS, patients are apt to notice and seek help for their symptoms from a health care professional. Therefore, it is possible to establish a definite time of onset of symptoms and follow patients through the acute and convalescent phases of ON. Furthermore, visual impairment can be quantified with reliable and validated measures of visual function including high- and low-contrast visual acuity, automated perimetry, and color vision testing [12–24]. Moreover, damage to the optic nerve causes atrophy of the RNFL, which can be measured and quantified with ocular imaging techniques, such as optical coherence tomography (OCT) [12–24]. The RNFL is the most proximal region of the afferent visual pathway, and it is a unique CNS structure because it lacks myelin. Given that the back of the eye represents the front of the brain, OCT provides noninvasive means to quantify the structural effects of an inflammatory insult to the optic nerve, which can then be compared to functional outcomes, to construct a structural-functional paradigm of CNS injury. For this paradigm to gain acceptance, however, OCT needs to provide a reliable means of detecting true pathological changes in the anterior visual pathway, which can be clearly distinguished from test-retest variability inherent to the technology. Furthermore, structural changes in the anterior visual pathway captured with OCT need to show concordance with other markers of disease activity in MS. As the data from OCT studies continue to mount, there may be evidence to support the tenability of the ON system model in clinical research and, potentially, to establish a role for OCT in the care of MS patients.

## 4. Optical Coherence Tomography

Optical coherence tomography (OCT) is a noninvasive, ocular imaging technique that uses low-coherence interferometry to generate in vivo, high-resolution (within 10 microns), cross-sectional images of the RNFL by measuring backscatter of infrared light [7, 25–28]. Early OCT systems employed a Michelson-type interferometer with a low-coherence-length, superluminescent diode light source [7]. One arm of the interferometer directed light onto the sample and collected the backscattered signal. A second reference arm had a reflecting mirror, which was mechanically controlled to vary the time delay and measure interference. The use of a low-coherence-length light source meant that interference occurred only when the distance traveled by the light in the sample and reference arms of the interferometer are matched to within the coherence length [7]. This characteristic allowed echo delays of the light from the tissue to be measured with temporal accuracy [7]. The data were processed and displayed as a two-dimensional, false-color image [7]. 

## 5. Early OCT Studies in Optic Neuritis: Breaking New Ground

The first study to investigate the role of OCT technology in the evaluation of MS patients was reported by Parisi [12], who used an early generation of OCT to compare RNFL values between 14 MS patients with prior ON and 14 age-matched controls. The thickness of the RNFL was 46% lower in MS eyes relative to control eyes (*P* < .01) and 28% lower in ON eyes as compared to unaffected eyes of the same patient (non-NON eyes) (*P* < .01) [7, 12]. In this paper, it was not clear whether patients had recurrent ON events, which may have contributed to the robust differences in RNFL thickness between ON eyes, non-ON eyes, and control eyes. Yet, even in the absence of known ON, RNFL values were 26% lower in MS eyes as compared to control eyes, which suggested that RNFL damage occurred independent of clinically overt ON in MS patients [7, 12]. Parisi's innovative work set the stage for followup studies, which would further delineate how changes in RNFL integrity are influenced by ON and illustrate how axonal changes in the anterior visual pathway mirror global CNS damage MS patients.

In a subsequent study, Trip and colleagues [13] compared RNFL values between 25 ON patients and 15 healthy controls. Optic neuritis patients were recruited with a selection bias towards incomplete visual recovery. Retinal nerve fiber layer thickness was significantly reduced (33%) in ON eyes (68.7 *μ*m) (*P* < .001) relative to control eyes (102.9 *μ*m) and in ON eyes (27%) (*P* < .001) relative to non-ON eyes (94.6 *μ*m) [13]. Retinal nerve fiber layer atrophy was associated with lower VEP amplitudes, worse logMAR visual acuity scores, reduced visual field mean deviation, and decreased color vision in ON patients [13]. This intriguing study further expanded our understanding of OCT by showing that, in addition to RNFL thickness, macular volumes were significantly reduced after ON in patients with incomplete visual recovery. More specifically, macular volumes were 11% in lower ON eyes as compared to control eyes (*P* < .001) and 9% lower in ON eyes relative to non-ON eyes (*P* < .001) [13]. 

In a more recent study, Burkholder et al. [14] further explored how inner and outer macular volumes related to RNFL thickness and visual function in 530 MS patients (with and without ON) and 111 control eyes. Lower macular volumes were associated with RNFL thinning, such that a 10-*μ*m difference in RNFL thickness corresponded to 0.20 mm^3^ reduction in total macular volume [14]. Correlations between RNFL thickness and inner macular volume were significant (*r* = 0.58, *P* < .  001), particularly in ON eyes relative to non-ON eyes (*r* = 0.61 versus *r* = 0.50) in MS patients [14]. These findings were significant because the ganglion cell layer comprises 34% of the total average macular thickness [14]; thus, tracking macular volumes in ON patients may help determine the temporal relation between primary neuronal cell death and axonal loss after a CNS inflammatory event. 

Fisher and colleagues [20] used OCT to compare RNFL values between 90 MS patients and 36 control subjects. While median Snellen acuity equivalents were better than 20/20 in both groups, mean RNFL thickness was reduced in MS patients (92 *μ*m) relative to controls (105 *μ*m) (*P* < .001) with the lowest values noted in the ON eyes (85 *μ*m) of MS patients (*P* < .001) [20]. Lower visual function scores were associated with reduced average overall RNFL thickness in MS patients, such that, for every 1-line decrease in low-contrast letter acuity or contrast sensitivity score, the mean RNFL thickness decreased by 4 *μ*m [20]. The findings of this study suggested a role for OCT as a structural biomarker and potential secondary outcome measure in future MS clinical trials. 

In 2006 [15], we reported the findings from 54 patients who were followed for a mean period of 13 months to determine whether the extent of RNFL thinning predicted visual recovery after acute ON. After a year, 74% of ON patients manifested significant RNFL atrophy in their affected eyes, with most RNFL loss occurring within 3 to 6 months of the ON event [15]. Average RNFL values were lower in ON eyes (78 *μ*m) relative to non-ON eyes (100 *μ*m) (*P* < .0001) [15]. Subclinical ON was detected in four patients during the course of the study [15]. These patients did not report a history of pain or vision loss, but ophthalmic testing showed a new visual field deficit, an abnormal visual-evoked potential (VEP) result, the evolution of optic disc pallor, and newly detected thinning of the RNFL to support the diagnosis of subclinical ON. By using regression analysis, we showed that there was a linear relationship between RNFL thickness and visual field mean deviation after ON, such that, below a “cutoff” of 75 *μ*m, every 10 *μ*m drop in RNFL was associated with a 6.46 dB decrease in mean sensitivity [15]. Our observations suggested that visual function may be relatively well preserved after ON until a critical threshold of axonal integrity is violated; after which, permanent vision loss is more likely to ensue.

Klistorner and colleagues [16] later evaluated 32 patients with unilateral ON and 25 control subjects with multifocal VEP (mfVEP) testing and OCT. The mean RNFL thickness in ON eyes (85 *μ*m) was reduced by 19.2% compared with control eyes (104 *μ*m) (*P* < .0001) [16]. There was a 39.8% reduction in the amplitude of the mfVEP in ON eyes relative to control eyes (*P* < .0001) [16]. Linear regression analysis demonstrated a strong correlation between inter-eye asymmetry values of RNFL thickness and mfVEP amplitude (*r* = 0.90, *P* < .0001). Lower RNFL values were also associated with increased mfVEP latency (*r* = −0.66, *P* < .002) [16]. In addition to demonstrating the utility of mfVEP in tracking optic nerve injury in ON patients, this study further confirmed the significant correlations between structural and functional measures of optic nerve integrity and showed that demyelination contributes to axonal loss in the anterior visual pathway.

## 6. Retinal Nerve Fiber Layer Atrophy: The Impact of Recurrent Optic Neuritis

After an isolated ON event, RNFL values decrease by approximately 20% when patients are recruited without selection bias [7, 15–20, 24, 29]. For eyes affected by two or more ON events, however, RNFL atrophy tends to be more severe [23, 30] and the corresponding impact on visual function, more dire. In a recent study of 193 MS patients, we compared RNFL values between 29 eyes affected by two or more ON events (recurrent ON eyes), 125 eyes affected by a single ON event (single ON eyes), and 232 non-ON eyes [23]. Retinal nerve fiber layer values were significantly lower in recurrent ON eyes (64.2 *μ*m) relative to single ON eyes (86.3 *μ*m ) (*P* < .0001) and non-ON eyes (100.1 *μ*m ) (*P* < .0001) [23]. Retinal nerve fiber layer atrophy was also significantly worse in single ON eyes as compared to non-ON eyes (*P* < .0001) [23]. Similarly, Yeh and colleagues [30] noted that average RNFL thickness decreased with increasing number of episodes of ON in pediatric patients. These findings indicate that recurrent inflammatory events have a cumulative impact and erode axonal integrity in the CNS.

**Figure 1:**
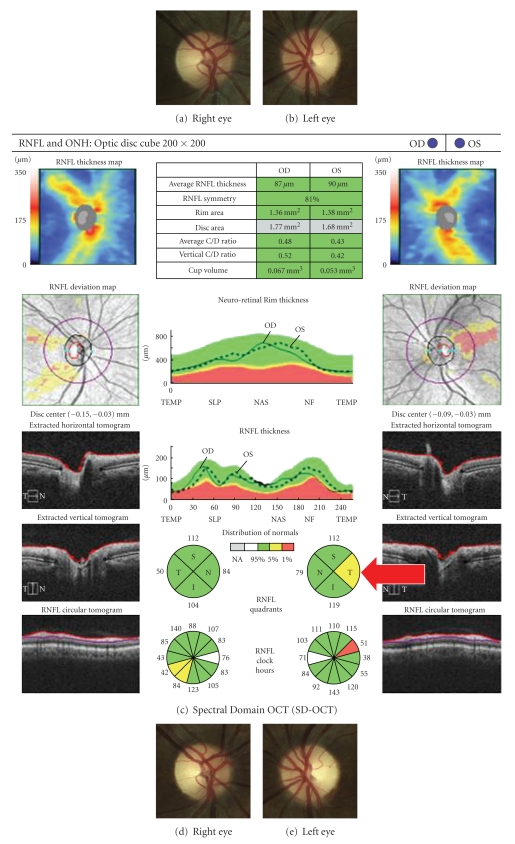

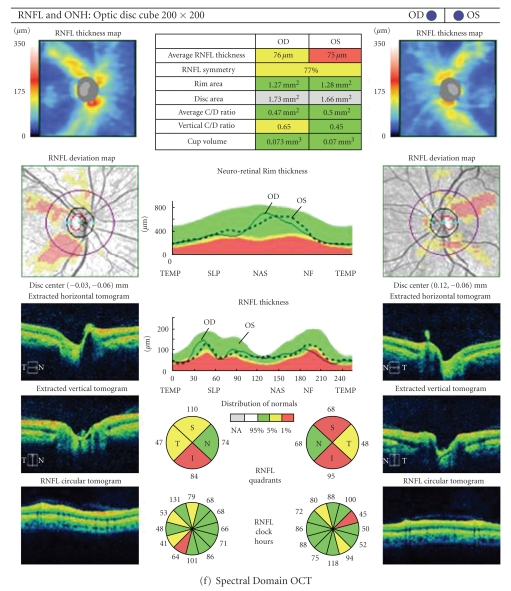
Case: A 26-year old woman with MS presented with a 2-month history of vision loss in both eyes. Best-corrected visual acuity was 20/150 in the right eye and count fingers (at 2 feet) in the left eye. There was a left relative afferent pupil defect. Fundus examination showed mild temporal pallor in the right (a) and left (b) eyes. Spectral domain OCT (c) showed that global average RNFL measurements were within normal limits in the right eye (OD) (87 *μ*m) and the left eye (OS) (90 *μ*m). There was relative temporal RNFL thinning in the left eye (arrow). Two and a half months later, the patient's visual acuity improved to 20/20 in both eyes, albeit with mild residual color vision deficits. There was more obvious temporal pallor in the right (d) and left (e) eyes. Repeat SD-OCT testing (f) showed progressive global average RNFL atrophy in the right (OD) (76 *μ*m) and left (OS) eyes (75 *μ*m).

The detection of new RNFL atrophy after ON can be more challenging in patients with prior ON as compared to patients experiencing their first ON event. Robust inter-eye differences in RNFL thickness may be observed for a CIS patient presenting with unilateral ON, because the anterior visual pathway has presumably been unscathed by prior inflammation. In contrast, a patient with RRMS may manifest less apparent inter-eye differences in RNFL thickness (Figure 1) and macular volume after ON if there has been previous optic nerve damage in the contralateral eye. Similarly, a patient with a new ON event and a prior history of ON in the same eye may show little change in RNFL thickness over time, because it is difficult to detect new RNFL thinning super imposed upon preexisting RNFL atrophy. Given the inherent heterogeneity of MS cohorts and the predilection for clinical and subclinical ON in this disease, caution is needed in the interpretation of RNFL values, particularly in cross-sectional studies.

## 7. Defining the Window of Axon Loss after Acute ON: Designing Future Clinical Trials

Only a few prospective studies have tried to define the time interval during which RNFL atrophy progresses after acute ON [17, 21, 24]. Establishing a potential therapeutic “window” is important for the design of future trials employing OCT as an outcome measure in ON patients. Noval and colleagues [21] followed 12 patients with acute ON and observed an initial increase in RNFL thickness, which resolved by 1.5 months. In ON eyes, they reported a 25% reduction in RNFL thickness at 6 months [21]. In 2008, we followed 78 ON patients over a mean period of 28 months and observed that the earliest significant difference in RNFL thickness between ON eyes and non-ON eyes manifested after two months, in the temporal RNFL region (inter-eye difference = 12.5 *μ*m, *P* = .005) [17]. In a subset of 20 patients who underwent regular OCT testing over a 12-month period, we reported that RNFL thinning progressed up to 6 months after acute ON and stabilized thereafter [17]. Similarly, in a recent longitudinal study, Henderson and colleagues [24] evaluated 23 patients with acute unilateral ON with serial OCT testing at presentation and after 3, 6, 12, and 18 months of follow up. Twelve control subjects were also imaged, on two occasions, a median of 552 days (range 350–907 days) apart [24]. Retinal nerve fiber layer values were significantly increased in ON eyes relative to non-ON eyes at baseline but then significantly decreased at all later time points [24]. Visual recovery at 12 months was not related to the extent of RNFL swelling seen acutely but was associated with the amount of RNFL loss observed in ON eyes [24]. As was noted in previous studies, the authors concluded that RNFL thinning is usually evident within 3 months of an acute ON event and that OCT-measured RNFL loss after 6 months is a tenable outcome measure for neuroprotection trials [24].

Given the small patient numbers included in the aforementioned studies, caution must again be exercised when interpreting the collective results. Our study included only 78 ON patients, and there was variability in followup across testing intervals [17]. To determine the time required for RNFL atrophy to stabilize within 12 months of an acute ON event, we tracked RNFL changes in a subset of 20 patients at regular intervals over a one-year period [17]. Our study lacked a control group, which further limited our conclusions [17]. Similarly, for the 23 ON patients and 12 control subjects studied by Henderson and colleagues there was some variability in followup [24]. It was also noteworthy that in ON eyes RNFL values ranged from 87 to 281 *μ*m (median 117 *μ*m, mean 133 *μ*m) at baseline, and two patients had initial RNFL values exceeding 200 *μ*m. Retinal nerve fiber layer values equal to or greater than 250 *μ*m are atypical in ON, because the extent of optic disc edema tends to be relatively mild relative to other optic neuropathies associated with more severe optic disc swelling (i.e., anterior ischemic optic neuropathy). When a patient population size is limited, it is unclear how outliers impact the overall interpretation of results, even in the context of an elegantly designed study. Therefore, further controlled, prospective clinical trials involving a larger numbers of patients will be needed to firmly establish the optimal time “window” to trial new therapeutic strategies in ON patients.

## 8. Optical Coherence Tomography: Risk of Multiple Sclerosis after Optic Neuritis

Only two prior studies have explored the association between RNFL atrophy and future risk of MS in ON patients, and the data were largely negative [18, 31]. Previously, we compared RNFL values in ON eyes and non-ON eyes between patients who developed CDMS (42%) and those that did not develop MS 24 months after an ON event (58%) [18]. Mean RNFL values were lower in ON eyes of non-MS patients as compared to CDMS ON eyes after one year (*P* = .05) due to more severe ON events in the former [18]. Temporal RNFL values were lower in the non-ON eyes of CDMS patients, but the results were not statistically significant (*P* = .13) [18]. From our findings, we concluded that RNFL thickness did not reliably distinguish patients at higher risk of converting to CDMS after ON. Similarly, Outteryck and colleagues [31] performed OCT testing on 56 CIS patients (18 with optic neuritis and 38 without optic neuritis) and 32 control subjects, to investigate whether measures of RNFL thickness and macular volume revealed early retinal axonal loss. In this prospective case series, there was no link between RNFL and (1) MRI evidence of disseminated CNS inflammation at baseline, (2) disseminated CNS inflammation according to the revised McDonald criteria, (3) gadolinium enhancement on initial MRI, (4) multifocal CIS presentation, (5) altered visual evoked potentials, or (6) development of “McDonald-” proven MS at 6 months [31]. Furthermore, patients who developed CDMS (*n* = 13) or McDonald-criteria proven MS (*n* = 23) did not have more severe RNFL atrophy [31]. These investigators concluded that OCT does not predict conversion to MS at 6 months in CIS patients and postulated that conversion to MS after ON is more likely influenced by inflammatory events than axonal degeneration.

## 9. Optical Coherence Tomography Studies in Pediatric Optic Neuritis Patients

Multifocal CNS demyelination has been reported to occur in approximately 0.4 per 100,000 of the pediatric patient population [30]. Similar to adults, ON is a relatively common occurrence in pediatric patients such that 22% of children experience ON as their first demyelinating event and 35% of children who eventually develop MS experienced ON during their first clinical episode [30]. In a recent pediatric ON study, Wilejto and colleagues [32] reported unilateral optic nerve involvement in the majority (58%) of pediatric patients (*n* = 36). Visual recovery after ON was considered complete in 39 of 47 affected eyes (83%) [32]. Cranial MRI scans demonstrated white matter lesions separate from the optic nerves in 54% of children. In this study, the risk of MS was 36% at 2 years, and bilateral ON was associated with a greater future risk of MS [32]. Clinical findings extrinsic to the visual system on baseline examination and MRI evidence of white matter lesions outside the optic nerves were strongly associated with a future diagnosis of MS [32]. Yeh and colleagues [30] used OCT in a cross-sectional study of 38 consecutive children (age <18 years) who had at least one documented clinical episode of an acquired demyelinating event and two control groups, including (1) 15 normal healthy children (30 eyes) with no history of neurological or other chronic disease and (2) 5 children (10 eyes) with other nondemyelinating disorders (OND), including headache, attention deficit hyperactivity disorder, and depression [30]. In MS patients RNFL thickness was 99 *μ*m in non-ON eyes and 83 *μ*m in ON eyes. Children with acute disseminated encephalomyelitis (ADEM) and transverse myelitis (TM) had lower RNFL values in ON eyes (67 *μ*m) relative to non-ON eyes (102 *μ*m) [30]. Macular volumes were markedly lower in ON eyes of children with ADEM/TM (6.2 mm^3^) and chronic relapsing inflammatory optic neuropathy (CRION) (6.0 mm^3^), suggesting a more widespread disease process in these clinical entities [30]. All subgroups with a clinical history of ON had lower average RNFL values (83 *μ*m for MS patients; 67 *μ*m for ADEM/TM patients; 89 *μ*m for CIS patients; 50 *μ*m for CRION patients) than controls (107 *μ*m). Differences between children with demyelinating disease and controls and between ON and non-ON eyes were statistically significant (*P* < .001). On the basis of their findings, the investigators concluded that OCT may be a valuable tool for monitoring anterior optic pathway dysfunction in children with demyelinating diseases.

## 10. Optical Coherence Tomography and Neuromyelitis Optica

Neuromyelitis optica (NMO) is a severe inflammatory process of the optic nerves and spinal cord and is associated with poor clinical recovery [33–38]. There have been several studies which have explored the role of OCT in quantifying the extent of axonal damage in the anterior visual pathway secondary to NMO with a view to distinguishing the ON associated with this clinical syndrome [36–38]. Naismith [36] used OCT to study 22 subjects with NMO or NMO spectrum disorders and 47 MS patients. In ON eyes, NMO was associated with lower RNFL values compared to MS, when controlling for visual acuity (57 *μ*m versus 67 *μ*m; *P* = .01) or for contrast sensitivity (61 *μ*m versus 70 *μ*m; *P* = .02). The superior and inferior quadrants were more severely affected in NMO than MS eyes. These authors noted that the odds of falling into the NMO group increased by 8% for every 1 *μ*m decrease in RNFL thickness [36]. Similar findings were noted by Ratchford and colleagues [37] who used OCT to study 26 NMO spectrum patients with a history of ON, 17 patients with isolated longitudinally extensive transverse myelitis (LETM) without ON, 378 patients with RRMS, and 77 healthy controls. These investigators observed significant RNFL thinning in NMO ON eyes (63.6 *μ*m) relative to RRMS ON eyes (88.3 *μ*m) (*P* < .0001) and control eyes (102.4 *μ*m) (*P* < .0001). A first episode of ON was estimated to cause 24 *μ*m more loss of RNFL thickness in NMO than RRMS eyes [37]. In a third study, Nakamura [38] evaluated 35 eyes of 18 patients with the “NMO spectrum” and 14 MS patients to determine whether RNFL thickness correlated with the clinical presentation in patients with NMO and ascertain what clinical factors lead to poor visual outcomes. Overall RNFL measurements were thinner in NMO ON eyes than MS ON eyes (64 *μ*m versus 84 *μ*m; *P* = .0006) especially in the superior and inferior RNFL quadrants [38]. Mean RNFL negatively correlated with the number of relapses in the NMO group. A receiver operating characteristic (ROC) analysis showed that the overall RNFL “cutoff” value for decreased visual acuity (measuring less than 20/20) was 71 *μ*m in the NMO group [38]. The frequency of the ON relapses and the time to initiate treatment with high-dose intravenous methylprednisolone significantly affected the preservation of RNFL thickness in this study [38]. Hence, the studies to date suggest that OCT may be used to distinguish anterior visual axis involvement in NMO from ON associated with MS.

## 11. Conclusions and Future Directions

Recent technological innovations have introduced the era of “Fourier” or “Spectral” domain OCT (SD-OCT). In this new generation of the device, all light echoes are detected simultaneously, leading to a dramatic increase in sensitivity that enables high-speed imaging [7]. Spectral domain OCT is now commercially available and provides an axial image resolution of 5–7 *μ*m, with imaging speeds of 25,000 axial scans per second. This imaging speed is approximately 50 times faster than the previous generations of OCT technology [7]. Retinal nerve fiber layer measurements in MS patients differ considerably between TD-OCT and SD-OCT devices, with excellent correlations between values obtained from both imaging techniques [39–43]. Recently, Bock and colleagues [39] compared SD-OCT and TD-OCT imaging techniques in 55 MS patients and reported a strong correlation (Pearson's *r* = 0.926, *P* < .001) between the two technologies. There were, however, significant differences in the absolute RNFL measurements (mean ± standard deviation 8.1 *μ*m ± 6.2, range −12 to 23 *μ*m), and therefore the results from the two devices were not interchangeable. The findings of this study were similar to those reported by Knight et al. [42] who compared SD-OCT and TD-OCT RNFL values in glaucomatous patients. In both studies, SD-OCT tended to measure “more thinly” than TD-OCT at higher RNFL values, whereas, for thinner RNFL values, SD-OCT measured “more thickly” than TD-OCT [39]. 

The reproducibility of SD-OCT retinal measurements relative to TD-OCT has recently been evaluated in MS and glaucoma patients. In a prospective study of 58 MS patients and 32 healthy controls, SD-OCT testing was performed to determine optimal intervisit, interrater, and intrarater reproducibility [41]. The authors noted excellent reproducibility of average and quadrant RNFL values, average macular thickness, and total macular volumes [41]. Leung and colleagues [40] evaluated RNFL measurement variability, diagnostic sensitivity and specificity, and the strength of the structure-function association obtained with SD-OCT versus TD-OCT in a prospective, cross-sectional study of 97 healthy controls and 83 glaucoma patients. The intra-visit repeatability of SD-OCT ranged between 5.12 and 15.02 *μ*m and the intervisit reproducibility, between 4.31 and 22.01 *μ*m. Overall, SD-OCT demonstrated lower measurement variability compared with TD-OCT. Finally, in a prospective observational study of 110 eyes, retinal measurements were compared between six different TD-OCT and SD-OCT devices including Stratus and Cirrus (Carl Zeiss Meditec, Inc.), Spectralis HRA + OCT (Heidelberg Engineering), RTVue-100 (Optovue Inc.), SDOCT Copernicus HR (Optopol Technology S.A.), and 3D OCT-1000 (Topcon Corporation) [43]. The standard analysis protocols for macular thickness evaluation were evaluated with each instrument. The six different devices produced measurements that differed in variance (Bartlett test, *P* = .006), and mean values (Friedman test, *P* < .001) [43]. Bland-Altman analyses showed that the limits of agreement for all the comparisons were not acceptable, and regression analysis revealed high standard error values [43]. The findings of this study indicated that retinal thickness measurements obtained with various OCT devices were different beyond clinical practice tolerance. The differences between devices were attributed to the analysis algorithms used to set retinal inner and outer boundaries [43].

Thus, while innovations in OCT technology offer potential advantages including minimizing error and improving test-retest reliability in the evaluation of MS patients, challenges remain. With each new reincarnation of the technology, there is a disruption in the collection of longitudinal data, which is vital in the management of a chronic disease. Furthermore, as the first SD-OCT studies are beginning to emerge, it is debatable whether any of these early reports have taught us anything that we did not already know regarding the potential role for OCT in the management of ON and MS patients. As the technology continues to advance, it is imperative that the results of OCT studies prove to be clinically relevant, not simply statistically significant to meaningfully impact our understanding and treatment of disease. In the context of MS, the challenge remains to define the amount of RNFL “signal” that represents pathology, and to distinguish this from the “noise” of the technology. This task may be onerous given the heterogeneity of MS cohorts and the prevalence subclinical disease activity in this patient population. On a practical level, MS patients may develop other occult ocular conditions (i.e., age-related macular degeneration and glaucoma) that damage the retinal architecture, making the interpretation of RNFL atrophy difficult. Also, retrograde transsynaptic retinal ganglion cell degeneration due to MS lesions within the posterior optic pathways has been shown to cause RNFL atrophy [44]. Thus, in the evaluation of RNFL thickness, it may also be necessary to distinguish the effects of postgeniculate lesions from subclinical disease in the anterior visual pathway in MS patients. 

In conclusion, there is increasing evidence to suggest a role for OCT in the evaluation of ON and MS patients. Optical coherence tomography may complement our existing arsenal of tools including tests of visual function, neuroimaging techniques, and electrophysiological studies and help develop a structural-functional paradigm of CNS inflammation. Furthermore, OCT may be used to capture structural changes in the anterior visual pathway, which will provide unique insights regarding pathogenic mechanisms of CNS injury and, in turn, to develop more effective therapeutic strategies for MS patients. Future longitudinal, large-scale studies will be needed to ultimately determine how this technology can be optimally implemented in a research setting, with the ultimate goal of enhancing patient care.
